# Differential Expression and Function of Arginase in Mouse Uterus During Early Pregnancy

**DOI:** 10.3390/ijms27104354

**Published:** 2026-05-14

**Authors:** Zai-Mei Wang, Qi-Man Shen, Hui-Na Luo, Hong-Yuan Yang, Jian Lu, Zeng-Ming Yang

**Affiliations:** 1 Key Laboratory of Animal Genetics, Breeding and Reproduction in the Plateau Mountain Region, College of Animal Science, Guizhou University, Guiyang 550025, China; 2 College of Veterinary Medicine, South China Agricultural University, Guangzhou 510642, China

**Keywords:** arginase, decidualization, implantation, oxidative stress

## Abstract

The supply of amino acids is essential to embryo survival and successful pregnancy. The accumulating evidence indicates that arginine, a semi-essential amino acid, plays a key role during early pregnancy. Arginase (ARG2) is a key enzyme for catalyzing arginine into ornithine and urea. However, the expression, regulation, and role of arginase during early mouse pregnancy are still unknown. In our study, ARG1 immunofluorescence is mainly detected in uterine epithelium and gradually decreases from days 1 to 5 of pregnancy. From days 1 to 4 of pregnancy, there is no detectable ARG2 immunofluorescence in the mouse uterus. On day 5 of pregnancy, ARG2 signals are strongly seen in the primary decidua surrounding the implanting blastocyst at the implantation site, but not at the inter-implantation site. There is a temporary increase for ARG2 levels under mouse in vitro decidualization, suggesting ARG2 may be involved in the initiation of mouse decidualization. *Prl8a2*, a marker of mouse in vitro decidualization, is significantly decreased after ARG levels are suppressed. However, *Arg2* overexpression obviously increases *Prl8a2* levels. Mouse in vitro decidualization is downregulated by arginine and ornithine but stimulated by a low dose of urea. Urea has a beneficial effect on uterine receptivity and antioxidative enzymes. Our results indicate that ARG2 plays an important role during mouse decidualization by balancing the levels of arginine, ornithine, and urea.

## 1. Introduction

Embryo implantation is the interaction between a competent embryo and a receptive uterus and a key step for successful pregnancy [1,2]. During decidualization, uterine stromal cells undergo morphological and functional changes into epithelial decidual cells [3]. Abnormal embryo implantation and decidualization lead to abnormal pregnancy outcomes [4,5]. However, the underlying mechanism of embryo implantation and decidualization still remains poorly defined.

Amino acids are essential for animal physiological activities, and their adequate supply is critical for organismal health, growth, development, and survival [6]. Based on dietary needs for growth, amino acids were traditionally classified as nutritionally essential or nonessential [7]. Amino acid uptake is an important part of cellular metabolism and is closely related to oocyte and early embryonic development [8]. Accumulating evidence indicates that an insufficient amino acid supply in sows during gestation leads to decreased embryonic survival in pigs [9,10]. Supplementation with amino acids during in vitro culture can significantly promote the development of bovine embryos [11]. The arginine supplement of female rats during early gestation or throughout pregnancy increases implantation sites and embryonic survival [12]. Dietary arginine supplementation during pregnancy in mice increased the total litter size, the number of live-born pups, the number of placental attachment sites, pup birth weight, and the body weight of live-born pups [13].

Arginine, a semi-essential amino acid, is the precursor for nitric oxide (NO) via nitric oxide synthase and for polyamines [14]. Arginine is derived from dietary intake and endogenous synthesis. It can be produced by the catalytic hydrolysis of argininosuccinate by argininosuccinate synthase 1 (ASS1) and argininosuccinate lyase (ASL) [15]. Arginine, one of the uterine histotrophs, is significantly increased in the uterine luminal lumen during the peri-implantation period [16,17]. By knocking down SLC7A1, the arginine transporter, it is shown that arginine is essential for conceptus survival and development [16]. The dietary supplement of arginine during early pregnancy can enhance embryo implantation in rats [18]. However, how arginine is regulated and how arginine acts during early pregnancy remain poorly defined.

Arginase (ARG), a key rate-limiting enzyme regulating arginine metabolism, catalyzes the hydrolysis of arginine to produce urea and ornithine [19,20]. There are two ARG isoforms in mammals: Arginase 1 (ARG1) and Arginase 2 (ARG2) [21,22]. ARG1 is mainly located in the liver and participates in the final step of the urea cycle, while ARG2 is a mitochondrial enzyme widely distributed in various tissues [23,24,25]. Ornithine is a substrate for polyamine synthesis. Polyamines are essential for male and female reproductive processes [26,27,28]. Although ARG1 and ARG2 are distinctly expressed in human endometrial epithelium during the menstrual cycle [29], the expression pattern and function of ARG1 and ARG2 in mouse uterus during early pregnancy are still unknown.

This study mainly investigated the expression and role of arginases in the mouse uterus during early pregnancy. Our data indicated that ARG2 is strongly expressed in decidual cells at the implantation site on day 5 of pregnancy and plays an essential role during mouse decidualization.

## 2. Results

### 2.1. ARG1 Immunofluorescence in Mouse Uterus During Early Pregnancy

**Figure 1:**
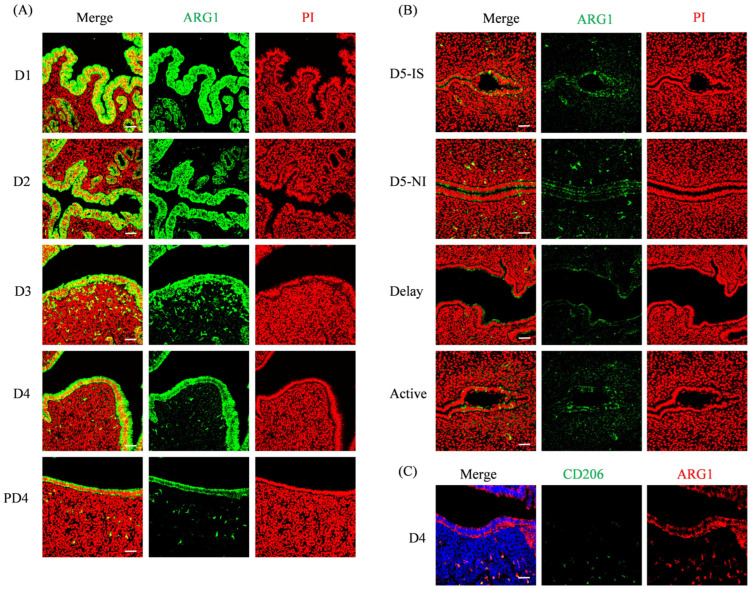
Expression of ARG1 in mouse uteri during early pregnancy. (**A**) ARG1 immunofluorescence in mouse uteri from days 1 (D1) to 4 (D4) of pregnancy and on day 4 of pseudopregnancy. (**B**) ARG1 immunofluorescence in mouse uteri at the implantation site (D5-IS) and non-implantation site (D5-NI) on day 5 of pregnancy, and under delayed implantation (Delay) and activation (Active), respectively. (**C**) Immunofluorescence colocalization of ARG1 and M2 macrophage marker CD206 on day 4 of pregnancy. Bar = 50 μm. n = 3 mice per group.

The ARG1 immunofluorescence signal was strongly localized in the uterine luminal epithelium and weakly in glandular epithelium on day 1 of pregnancy and gradually decreased from days 1 to 4 of pregnancy. On day 4, the ARG1 signal was mainly detected in the luminal epithelium. Compared to day 4 of pregnancy, the ARG1 signal in the luminal epithelium was weaker on day 4 of pseudopregnancy (Figure 1A). On day 5 of pregnancy, the ARG1 signal was weakly detected in the luminal epithelium and in the subluminal stroma at the implantation site, and was only weakly seen in the luminal epithelium. Under delayed implantation, there was a weak ARG1 signal in luminal epithelium. After delayed implantation was activated by estrogen, the ARG1 signal was weakly observed in both the luminal epithelium and the subluminal stroma (Figure 1B).

Additionally, the ARG1 signal was also diffusely seen in the uterine stroma from days 1 to 5 of pregnancy (Figure 1A,B). It is shown that ARG1 is also a marker for M2 macrophages [30]. Macrophages show distinct functional phenotypes under different inflammatory signals and are classified into inflammatory M1 (MHC II-positive) and anti-inflammatory M2 subtypes (CD206-positive) [31,32]. We demonstrated that ARG1 signals were co-localized with the M2 macrophage marker CD206 in the day 4 pregnant uterus (Figure 1C). These ARG1-positive cells in the uterine stroma are likely M2 macrophages.

### 2.2. ARG2 Immunofluorescence in Mouse Uterus During Early Pregnancy

**Figure 2:**
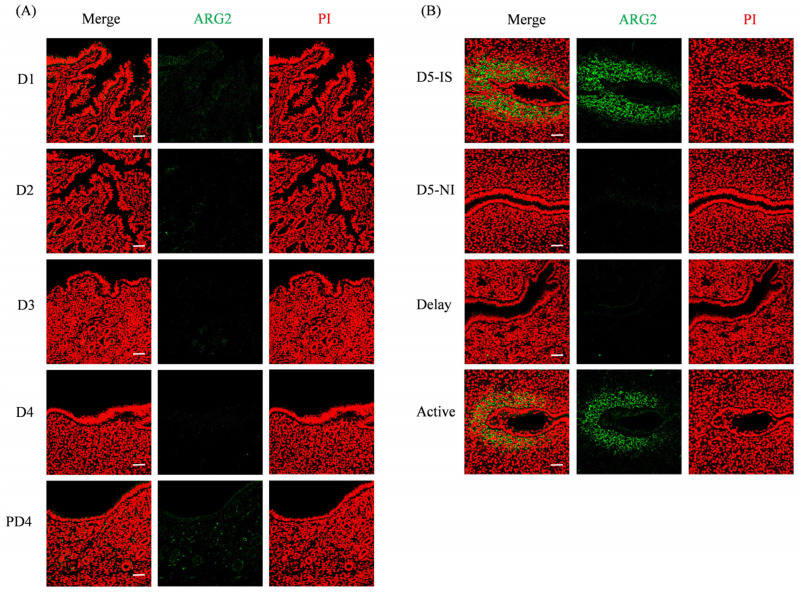
ARG2 immunofluorescence in mouse uteri during early pregnancy. (**A**) ARG2 immunofluorescence from days 1 (D1) to 4 (D4) of pregnancy, and on day 4 of pseudopregnancy. (**B**) ARG2 immunofluorescence in mouse uteri at the implantation site (D5-IS) and non-implantation site (D5-NI) on day 5 of pregnancy, and under delayed implantation (Delay) and activation (Active), respectively. Bar = 50 μm. n = 3 mice per group.

From days 1 to 4, there was no detectable ARG2 fluorescence signal in mouse uteri. ARG2 was also not detected in the mouse uterus on day 4 of pseudopregnancy (Figure 2A). On day 5 of pregnancy, ARG2 signals were clearly observed in the subluminal stromal cells surrounding the implanting blastocyst at the implantation site, but were not seen at the non-implantation site. Under delayed implantation, there was no detectable ARG2 signal in the mouse uterus. After delayed implantation was terminated by estrogen, the ARG2 signal was similar to that at the implantation site on day 5 of pregnancy (Figure 2B).

### 2.3. The Role of ARG2 in Decidualization

**Figure 3:**
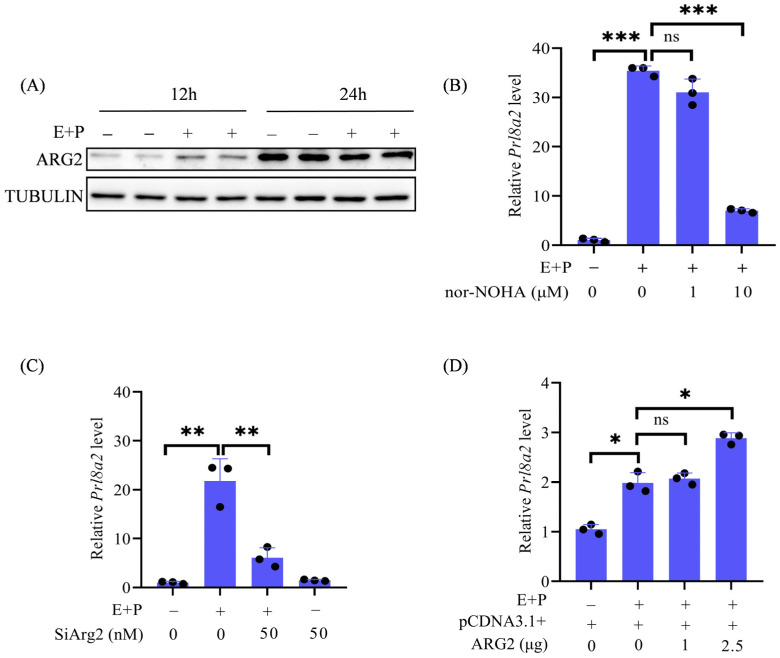
The role of ARG2 during in vitro decidualization. (**A**) The levels of ARG2 protein during in vitro decidualization for 12 and 24 h. (**B**) The levels of *Prl8a2* mRNA in mouse endometrial stromal cells treated with different concentrations of nor-NOHA. (**C**) The *Prl8a2* mRNA level after stromal cells under in vitro decidualization were transfected with *Arg2* siRNA for 24 h. (**D**) The level of *Prl8a2* mRNA after mouse stromal cells under in vitro decidualization were overexpressed with *Arg2*. All images are representative of at least three biologically independent experiments. ns: not significant; * *p* < 0.05; ** *p* < 0.01; *** *p* < 0.001.

Because ARG2 was strongly detected in the subluminal stromal cells at the implantation site located in the primary decidual zone, we would like to explore whether ARG2 is involved in decidualization. Under in vitro decidualization, ARG2 protein levels were higher at 12 h but lower at 24 h compared to control (Figure 3A). Nomega-hydroxy-nor-L-arginine (nor-NOHA) is a competitive inhibitor [33]. *Prl8a2* is a marker of mouse in vitro decidualization [34]. Compared to control, the *Prl8a2* mRNA level was significantly increased under in vitro decidualization, which was significantly abrogated by 10 μM nor-NOHA (Figure 3B). To investigate the specific role of ARG2 during decidualization, *Arg2* expression was inhibited by *Arg2* siRNA. The *Prl8a2* mRNA level under in vitro decidualization was also significantly suppressed by *Arg2* siRNA (Figure 3C). To further examine the role of ARG2 during decidualization, the *Prl8a2* mRNA level was significantly enhanced by *Arg2* overexpression (Figure 3D). These results indicated that ARG2 is beneficial for mouse in vitro decidualization.

### 2.4. The Effects of Arginine, Ornithine, and Urea on Decidualization

**Figure 4:**
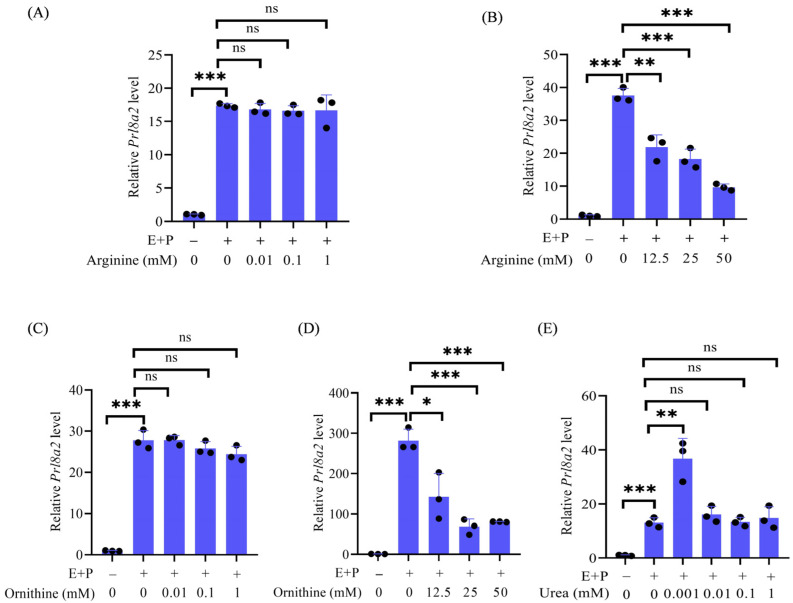
Effects of arginine, ornithine, and urea on in vitro decidualization. (**A**) Effects of low concentrations of arginine (0.01, 0.1, and 1 mM) on *Prl8a2* level. (**B**) Effects of high concentrations of arginine (12.5, 25 and 50 mM) on *Prl8a2* level. (**C**) Effects of low concentrations of ornithine (0.01, 0.1, and 1 mM) on *Prl8a2* level. (**D**) Effects of high concentrations of ornithine (12.5, 25, and 50 mM) on *Prl8a2* level. (**E**) Effects of urea on *Prl8a2* level. All images are representative of three biologically independent experiments. ns: not significant; * *p* < 0.05; ** *p* < 0.01; *** *p* < 0.001.

Because arginase catalyzes the production of urea and ornithine from arginine [19,20], we examined the roles of arginine, urea, and ornithine during in vitro decidualization, respectively. Under in vitro decidualization for 24 h, the low concentrations (0.01, 0.1, and 1 mM) of arginine had no effects on *Prl8a2* mRNA expression (Figure 4A), whereas the high concentrations (12.5, 25, and 50 mM) of arginine significantly inhibited *Prl8a2* mRNA expression in a dose-dependent manner (Figure 4B). As a downstream product, ornithine at the low concentrations (0.01, 0.1, and 1 mM) showed no effects on *Prl8a2* mRNA expression (Figure 4C), but the high concentrations (12.5, 25, and 50 mM) significantly inhibited *Prl8a2* mRNA expression (Figure 4D). However, another downstream metabolite of arginine, 1 μM urea, significantly increased *Prl8a2* mRNA expression under in vitro decidualization (Figure 4E). These results indicated that a low dose of urea could promote mouse in vitro decidualization.

### 2.5. The Influence of Arginine, Ornithine, and Urea on Uterine Receptivity

**Figure 5:**
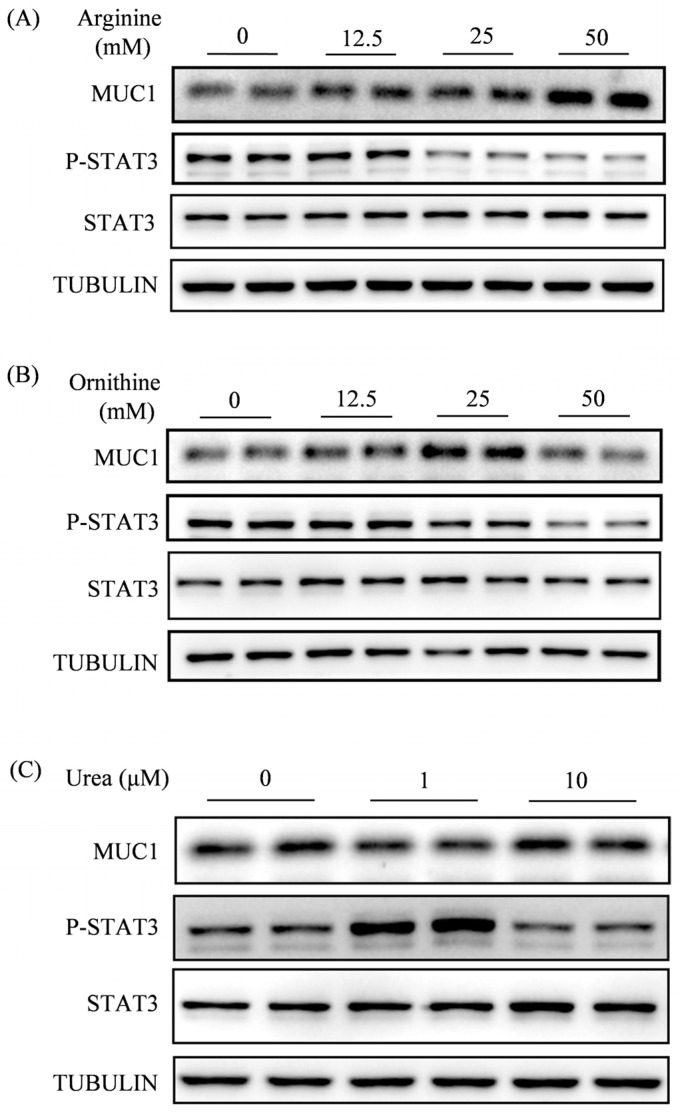
Effects of arginine, ornithine, and urea on uterine receptivity. (**A**) Western blot analysis and quantification of MUC1 and p-STAT3 protein levels in mouse uterine epithelial cells treated with arginine. (**B**) Western blot analysis and quantification of MUC1 and p-STAT3 protein levels in mouse uterine epithelial cells treated with ornithine. (**C**) Western blot analysis and quantification of MUC1 and p-STAT3 protein levels in mouse uterine epithelial cells treated with urea. All images are representative of three biologically independent experiments.

Given that ARG1 was mainly localized in the luminal epithelium on 4 of pregnancy, we would like to examine effects of arginine, ornithine, and urea on uterine receptivity, respectively. Endometrial receptivity, a prerequisite for embryo implantation, is characterized by the downregulation of Mucin 1(MUC1) and the upregulation of phosphorylated signal transducer and activator of transcription 3 (p-STAT3) in luminal epithelium on day 4 of pregnancy [35,36]. After mouse uterine epithelial cells were treated with arginine, 12.5 mM arginine had no effects on both p-STAT3 and MUC1 levels, but 25 mM or 50 mM arginine significantly decreased p-STAT3 levels and increased MUC1 levels (Figure 5A). When epithelial cells were treated with ornithine, 12.5 mM ornithine had no effects on both p-STAT3 and MUC1, but 25 mM and 50 mM ornithine significantly suppressed p-STAT3 levels, and only 25 mM ornithine significantly increased MUC1 levels (Figure 5B). For urea treatments, 10 μM had no effects on both p-STAT3 and MUC1 levels, but 1 μM urea significantly increased p-STAT3 levels and reduced MUC1 levels (Figure 5C). These results indicated that endometrial receptivity could be enhanced by 1 μM urea.

### 2.6. Arginine, Ornithine, and Urea Cause Abnormal Expression of Oxidative Stress Molecules During Decidualization

**Figure 6:**
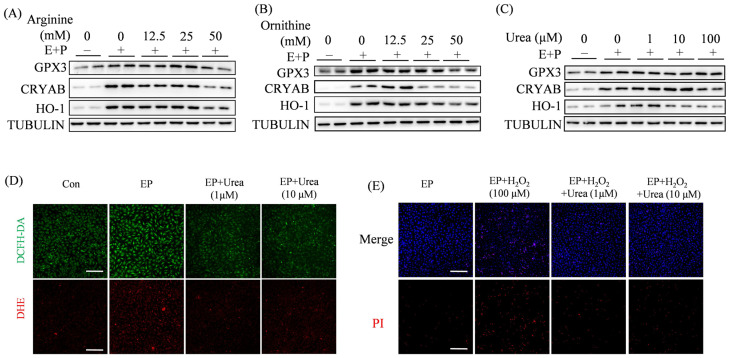
Effects of arginine, ornithine, and urea on antioxidative enzymes. (**A**) Western blot analysis and quantification of GPX3, HO1, and CRYAB protein levels when mouse stromal cells were treated with arginine under in vitro decidualization. (**B**) Western blot analysis and quantification of GPX3, HO1, and CRYAB protein levels when mouse stromal cells were treated with ornithine under in vitro decidualization. (**C**) Western blot analysis and quantification of GPX3, HO1, and CRYAB protein levels when mouse stromal cells were treated with urea under in vitro decidualization. (**D**) Effects of urea on DCFH-DA fluorescence (green) and DHE fluorescence (red) in mouse endometrial stromal cells treated with urea under in vitro decidualization for 12 h. Scale bar = 100 μm. (**E**) Effects of urea on cell viability (dead cells were stained by PI as red fluorescence) after H_2_O_2_-treated mouse endometrial stromal cells were co-treated with urea for 12 h under in vitro decidualization. Scale bar = 100 μm. All images are representative of three biologically independent experiments.

During decidualization, stromal cells face the challenges from oxidative stress [37]. Heme oxygenase-1(HO-1), Crystallin αB (CRYAB), and glutathione peroxidase 3 (GPX3) are important antioxidant molecules and highly expressed during decidualization [35,38,39]. Because arginine is able to reduce oxidative stress and inflammation [40], we would like to see whether arginine, ornithine, and urea have any effects on antioxidant enzymes. Under in vitro decidualization, HO-1, CRYAB, and GPX3 were significantly increased. Arginine had no effects or inhibitory effects on HO-1, CRYAB, and GPX3 (Figure 6A). Ornithine also showed inhibitory effects on HO-1, CRYAB, and GPX3, except that 12.5 mM ornithine had a beneficial effect on CRYAB (Figure 6B). Unlike arginine and ornithine, 1 μM urea significantly increased the levels of HO-1 and CRYAB; 10 μM urea only upregulated the CRYAB level, but 100 μM urea had no effects on either HO-1 or CRYAB (Figure 6C). Additionally, three doses of urea had no obvious effects on GPX3.

In this study, fluorescent probes DCFH-DA and DHE are used to show the levels of ROS and the superoxide, respectively. Compared to the control, the fluorescence intensity of DCFH-DA and DHE was increased under in vitro decidualization, which was abrogated by urea treatment (Figure 6D). Hydrogen peroxide (H_2_O_2_) is a classic ROS inducer. Compared to the control, H_2_O_2_ treatment caused an obvious increase of dead cells in uterine stromal cells under in vitro decidualization, which was suppressed by either 1 μM or 10 μM urea (Figure 6E). These results indicated that urea inhibits ROS production and effectively rescues H_2_O_2_-induced stromal cell death.

### 2.7. Effects of Arginine, Ornithine, and Urea on ARG2

**Figure 7:**
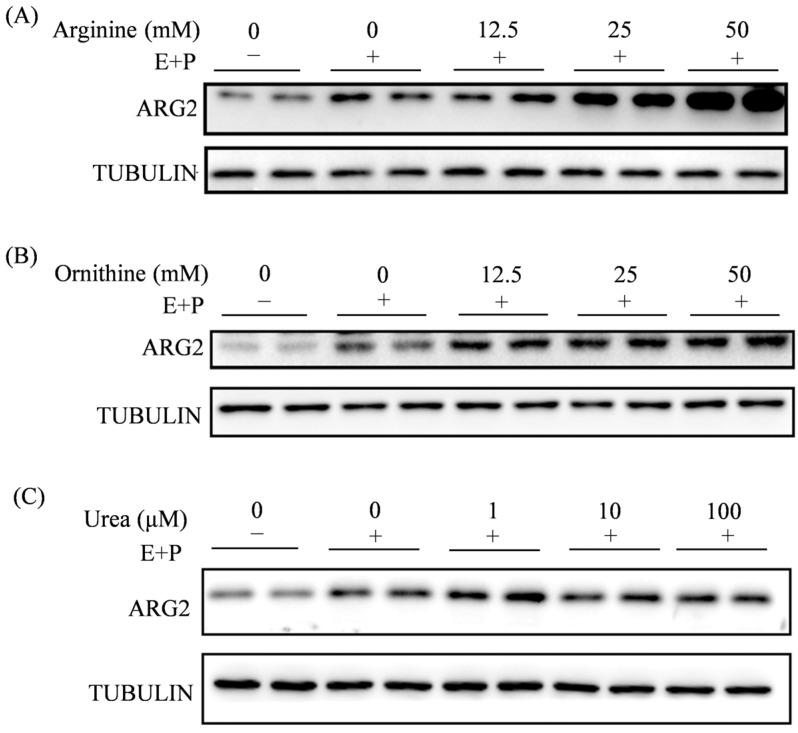
Effects of arginine, ornithine, and urea on ARG2. (**A**) Western blot analysis and quantification of ARG2 protein levels when mouse stromal cells were treated with arginine during in vitro decidualization. (**B**) Western blot analysis and quantification of ARG2 protein levels when mouse stromal cells were treated with ornithine during in vitro decidualization. (**C**) Western blot analysis and quantification of ARG2 protein levels when mouse stromal cells were treated with urea during in vitro decidualization. All images are representative of three biologically independent experiments.

Given that ARG2 is responsible for the production of ornithine and urea from arginine, we would like to explore whether arginine, ornithine, and urea have any effects on ARG2 during decidualization. Under in vitro decidualization for 12 h, ARG2 was significantly promoted. Arginine significantly increased ARG2 levels in a dose-dependent manner (Figure 7A). Ornithine had an enhancing effect on ARG2 levels (Figure 7B). However, ARG2 levels were upregulated by 1 μM urea, but 10 and 100 μM urea had no effect on ARG2 levels (Figure 7C). These results indicated that ARG2 was positively regulated by arginine, ornithine, and urea.

## 3. Discussion

This study demonstrates that arginase plays a significant role in mouse embryo implantation and decidualization and regulates the decidualization process through the joint action of arginine, ornithine and urea.

Arginase is the key enzyme in the catabolism of arginine, which can catabolize arginine to generate ornithine and urea [41,42]. Ornithine is the main precursor of polyamines and proline, and polyamines and proline are essential for cell proliferation and collagen synthesis. The deficiency of ARG1 will affect the urea cycle in the liver, leading to hyperargininemia with spastic paraplegia, progressive neurological and intellectual impairment, persistent growth retardation, and hyperammonemia [43]. Abnormal accumulation of arginine may harm fetal development [44]. During pregnancy, embryo implantation requires the uterus to transition into a receptive state. Failure to achieve this state may impede blastocyst attachment and result in impaired pregnancy outcomes [45]. In the human uterus, ARG1 and ARG2 are localized in human endometrial epithelial cells during both the proliferative and secretory phases, and the expression level of ARG2 is significantly higher in the secretory phase than in the proliferative phase [29]. Our results showed that ARG1 was localized in the uterine epithelium of the mouse uterus from days 1 to 4 of pregnancy and strongly localized in uterine stromal cells. These ARG1-positive cells in mouse stroma should be macrophages. ARG1 is expressed in M2 macrophages [30]. Macrophages are abundantly distributed in the preimplantation mouse uterus [32]. Phosphorylated STAT3 is highly localized in luminal epithelium on day 4 of pregnancy [46]. In human myeloid-derived suppressor cells, signal transducer and activator of transcription 3 (STAT3) transcriptionally regulates ARG1 expression [47]. It is possible that ARG1 expression in mouse uterine epithelium may be transcriptionally regulated by STAT3.

In our study, ARG2 is mainly localized in decidual cells at the implantation site on day 5 of pregnancy, suggesting that ARG2 should be related to decidualization. By either suppressing or overexpressing *Arg2*, we showed that ARG2 plays a key role during mouse in vitro decidualization. Among two metabolites of arginine (ornithine and urea), urea also shows a beneficial effect on mouse in vitro decidualization. Regarding the role of ARG2 during embryo implantation and decidualization, there are contradictory data. When pregnant rats are dietarily supplemented with arginine, embryonic survival and embryo implantation are enhanced [18,48]. In ungulates like pigs and sheep, arginine concentration increases in the uterine lumen during the peri-implantation period and shows a beneficial role for conceptus survival and development [16,49]. However, a dietary supplement of 0.8% L-arginine between days 0 and 25 of pregnancy reduces littler size in gilts [50]. In our study, the low dose of arginine has no obvious effect on mouse in vitro decidualization, but the high dose of arginine shows an inhibitory effect on both in vitro decidualization and uterine receptivity. It is possible that a certain range of physiological arginine should be beneficial for early pregnancy. In human lung endothelial cells, ARG2 expression is regulated by HIF2 under hypoxia [51]. In the mouse uterus, HIF2α is strongly expressed in decidual cells surrounding the implanting blastocyst on day 5 of pregnancy [52], similar to the ARG2 expression pattern. It is possible that ARG2 is regulated by HIF2α.

MUC1 is a transmembrane glycoprotein that is downregulated at the apical surface of the uterine luminal epithelium during embryo implantation, serving as a marker molecule for the non-receptive state of the uterus [53,54]. Our results demonstrated that treatment with high concentrations of arginine and ornithine upregulates MUC1 expression in mouse epithelial cells, whereas urea treatment downregulates MUC1 expression. STAT3 phosphorylation at tyrosine 705 in uterine epithelial cells is necessary for embryo implantation, serving as a marker of uterine receptivity [55]. Our results demonstrated that treatment with high concentrations of arginine and ornithine downregulates the level of p-STAT3 in mouse epithelial cells, whereas urea treatment upregulates p-STAT3 expression. These findings further suggest that high concentrations of arginine and ornithine may impair endometrial receptivity, whereas urea exerts a beneficial effect.

Arginine is shown to have a reduction in oxidative stress and inflammation [40]. Exogenous L-arginine administration also shows a beneficial effect on heat stress in pregnant buffaloes under subtropical conditions [56]. The antioxidant enzymes (CRYAB, HO-1, and GPX3) are highly expressed in decidual cells at implantation sites [57,58,59], which shows a similar pattern to ARG2. In our study, only urea has a slightly beneficial effect on HO-1 and CRYAB.

## 4. Materials and Methods

### 4.1. Animals and Treatments

Mature ICR mice (6–8 weeks old) were purchased from Hunan Sileke Jingda Laboratory Animal Co., Ltd. in Changsha, China, and were maintained in a temperature-controlled environment with a 12 h light cycle. All animal protocols were approved by the Animal Care and Use Committee of Guizhou University (EAE-GZU-2023-T005). Pregnant and pseudopregnant female mice were obtained by mating with fertile or vasectomized male mice, respectively. The day when the vaginal plug was seen was defined as day 1 of pregnancy (D1) or pseudopregnancy. The implantation site was determined on day 5 by intravenous injection of 0.2 mL of 1% Chicago blue dye (Sigma-Aldrich, St. Louis, MO, USA) dissolved in normal saline.

### 4.2. Delayed Implantation and Activation Model

As mentioned before [60], mice on day 4 of pregnancy were subjected to bilateral ovariectomy before 10:00 a.m. and subcutaneously injected with progesterone (1 mg/0.1 mL per mouse) in the morning from days 5 to 6 of pregnancy to maintain the state of delayed implantation. On day 7 of pregnancy, ovariectomized mice were divided into two groups for treatment: one group continued to receive a subcutaneous injection of progesterone to maintain the delayed implantation state, and the other group received a subcutaneous injection of progesterone and estradiol (E2, 25 ng/0.1 mL per mouse) to activate embryo implantation [60]. On the morning of day 8 of pregnancy, mice in the delayed implantation group were sacrificed to collect uterine tissues after blastocysts hatched from the zona pellucida were flushed from one side of the uterus to confirm delayed implantation. When delayed implantation was terminated by estrogen treatment, implantation sites and inter-implantation sites were collected after mice were intravenously injected with 0.2 mL of 1% Chicago blue dye.

### 4.3. Immunofluorescence

Immunofluorescence was performed as previously described [61]. The uterine tissues were fixed in 4% neutral buffered formalin, dehydrated through an alcohol gradient, and embedded in paraffin. The paraffin sections were dewaxed, rehydrated, and antigen retrieved through boiling in citrate buffer (pH 6.0) or Tris/EDTA buffer (pH 9.0). Sections were blocked with a 10% horse serum and incubated with the corresponding primary antibody at 4 °C overnight. The primary antibodies used in this study included ARG1 (1:200, 16001-1-AP, Proteintech, Wuhan, China) and ARG2 (1:500, 55003S, Cell Signaling Technology, Danvers, MA, USA). After three washes with PBS, sections were incubated with matched secondary antibodies conjugated with FITC (2.5 μg/mL, G21234, Invitrogen, Carlsbad, CA, USA) for 30 min at 37 °C, counterstained with propidium iodide (5 g/mL, PI, P4170, Sigma-Aldrich), and mounted with ProLong Diamond Antifade Mountant (Thermo Fisher’s, Waltham, MA, USA). Fluorescence signals were captured using a Nikon C2 confocal microscope (Nikon, Tokyo, Japan).

### 4.4. Western Blot

Western blot was performed as previously described [62]. After the cultured cells were lysed with RIPA (R0010, Solarbio, Beijing, China), the protein concentration was determined by the BCA method (23,225, Thermo Fisher Scientific, Waltham, MA). Protein samples were separated by SDS-polyacrylamide gel electrophoresis and transferred onto PVDF membranes (Immobilon^®^-P, IPVH00010, Millipore, Billerica, MA, USA). After being blocked with 5% nonfat milk (A600669, Sangon Biotech, Shanghai, China), the PVDF membranes were incubated with each primary and secondary antibody, respectively. The signal was detected using the ECL chemiluminescence kit (Millipore). The primary antibodies used in this study included ARG2 (1:1000, 55003S, Cell Signaling Technology), CRYAB (1:1000, ab281561, Abcam, Cambridge, UK), HO-1(1:1000, 10701-1-AP, Proteintech), GPX3 (1:1000, ab256470, Abcam), MUC1 (ab45167, Abcam), p-STAT3 (1:1000, ab76315, Abcam, Cambridge, UK), STAT3 (1:1000, 9139 s, Cell Signaling Technology), and TUBULIN (1:1000, 2144 S, Cell Signaling Technology).

### 4.5. Isolation and Treatment of Mouse Uterine Luminal Epithelial Cells

Uterine luminal epithelial cells were isolated as previously described [63]. Mouse uteri from day 4 pseudopregnant mice were longitudinally cut, rinsed in HBSS three times and incubated in the digestion solution (0.2% trypsin, 6 mg/mL Dispase, 4.3 mL HBSS, and 50 μL streptomycin/penicillin) at 4 °C for 1.5 h, room temperature for 30 min, and 37 °C for 10 min. Following three rinses in HBSS, cells were cultured with DMEM/F12 containing 10% FBS (040011A, Biological Industries, Cromwell, CT, USA) for 30 min. Then the unattached epithelial cells were transferred into new culture plates precoated with ECM (1:100, E0282, Sigma-Aldrich) for further culture. To study the effects of arginine, ornithine, and urea on the receptive state of mouse uterine epithelium, different concentrations of arginine (A8094, Sigma-Aldrich), ornithine (O2375, Sigma-Aldrich), and urea (PHR1406, Sigma-Aldrich) were used to treat them, respectively.

### 4.6. Isolation and Treatment of Mouse Endometrial Stromal Cells

After the epithelial cells were isolated, the remaining uterine tissue was further digested in HBSS containing 0.15 mg/mL type I collagenase (17100-017, Invitrogen, Houston, TX, USA) at 37 °C for 35 min. Stromal cells were collected and cultured in DMEM/F12 medium containing 10% FBS (D2906, Sigma-Aldrich, St. Louis, MO, USA). Mouse stromal cells were induced for in vitro decidualization using 10 nM E2 (HY-B0141, MedChemExpress, Monmouth Junction, NJ, USA) and 1 μM P4 as previously described [64]. *Prl8a2* is a marker of mouse in vitro decidualization [65]. To investigate the effects of arginine, ornithine, and urea on decidualization, mouse stromal cells were treated with different concentrations of arginine (A8094, Sigma-Aldrich), ornithine (O2375, Sigma-Aldrich), and urea (PHR1406, Sigma-Aldrich).

### 4.7. Real-Time RT-qPCR

Real-time quantitative reverse transcription polymerase chain reaction (RT-qPCR) was performed as previously described [66]. Total RNAs were extracted from mouse stromal cells using TRIzol (AG21101, Accurate Biology, Changsha, China), digested with RQ1 deoxyribonuclease I (Promega, Fitchburg, WI, USA), and reverse-transcribed into cDNA with the Prime Script Reverse Transcriptase Reagent Kit (Takara, Japan). RT-qPCR was performed using the SYBR Premix (Q311-02-AA, Vazyme, Nanjing, China). The data were analyzed using the 2^−ΔΔCt^ method and normalized to mouse *Rpl7*. Primers were designed and synthesized by Shanghai Sangon Biotech Co., Ltd. (Shanghai, China). All primer sequences utilized for RT-qPCR were provided in Table 1.

### 4.8. Transfection of Overexpression Plasmids

The *Arg2* overexpression plasmid was purchased from Beijing Liuhe Huada Gene Technology Co., Ltd. Mouse uterine stromal cells were transfected with 2.5 μg of the *Arg2* plasmid or empty vector using Lipo2000 (11,668,019, Invitrogen). The control was transfected for 6 h and further cultured in DMEM/F12 with 10% cFBS for 12 h or 24 h.

### 4.9. Transfection of Small Interfering RNA

The *Arg2* interference fragments were designed and purchased from Beijing Tsingke Biotechnology Co., Ltd. Three interference fragments of *Arg2* were constructed and transfected into stromal cells using Lipo2000 at different concentrations. The interference efficiency was determined by real-time qPCR. It was found that the interference fragment *siArg2*-2 had the most significant interference efficiency at 24 h. Therefore, *siArg2*-2 was used for further study.

### 4.10. Detection of Reactive Oxygen Species and Cell Viability

As previously described [67,68], Dihydroethidium (DHE) and 2′,7′-Dichlorodihydrofluorescein diacetate were used to monitor ROS levels in mouse endometrial stromal cells treated with 1 μM and 10 μM urea for 12 h. DCFH-DA (10 μM, D6470, Solarbio, Beijing, China) and DHE (10 μM, 50102ES25, Yeasen Biotechnology, Shanghai, China) were incubated at 37 °C for 30 min in the dark for ROS detection. Propidium iodide (PI) is a fluorescent dye that can enter the cell to stain nuclear DNA when the integrity of the membrane is damaged [69]. One hundred μM H_2_O_2_-treated mouse endometrial stromal cells were co-treated with urea for 12 h under in vitro decidualization, followed by incubation with PI (5 g/mL, P4170, Sigma-Aldrich) at 37 °C for 5 min in the dark for cell death detection.

### 4.11. Statistical Analysis

The data were analyzed using GraphPad Prism 9.0 software. Student’s *t*-test was used to examine the differences between the two groups. One- or two-way analysis of variance (ANOVA) test was used to compare multiple groups. Data were presented as mean ± SD (N = 3 biologically independent experiments). Statistical significance was defined as *: *p* < 0.05; **: *p* < 0.01; ***: *p* < 0.001. ns: not significant.

## 5. Conclusions

Our results indicate that ARG1 and ARG2 are differentially expressed in mouse peri-implantation uterus and play an important role during mouse embryo implantation and decidualization by balancing the levels of arginine, ornithine, and urea. These findings should be beneficial for improving reproductive health and optimizing pregnancy outcomes.

## Figures and Tables

**Table 1:** RT-qPCR primer sequences.

Genes	Species	Sequence (5′-3′)	Accession Number
*Prl8a2*	Mouse	AGCCAGAAATCACTGCCACT TGATCCATGCACCCATAAAA	NM_010088
*Rpl7*	Mouse	GCAGATGTACCGCACTGAGATTC ACCTTTGGGCTTACTCCATTGATA	NM_011291.5

## Data Availability

The original contributions presented in this study are included in the article. Further inquiries can be directed to the corresponding author.

## References

[1] Zhang S., Lin H., Kong S., Wang S.B., Wang H.M., Wang H.B., Armant D.R. (2013). Physiological and Molecular Determinants of Embryo Implantation. Mol. Asp. Med..

[2] Ojosnegros S., Seriola A., Godeau A.L., Veiga A. (2021). Embryo Implantation in the Laboratory: An Update on Current Techniques. Hum. Reprod. Update.

[3] Huang Y.F., Zhu Q.L., Sun Y. (2025). Glucose Metabolism and Endometrium Decidualization. Front. Endocrinol..

[4] Chen Q., Zhang Y., Peng H.Y., Lei L., Kuang H.B., Zhang L., Ning L., Cao Y.J., Duan E. (2011). Transient {beta}2-Adrenoceptor Activation Confers Pregnancy Loss by Disrupting Embryo Spacing at Implantation. J. Biol. Chem..

[5] Gu X.-W., Yang Y., Li T., Chen Z.-C., Fu T., Pan J.-M., Ou J.-P., Yang Z.-M. (2020). ATP Mediates the Interaction between Human Blastocyst and Endometrium. Cell Prolif..

[6] Ren W.K., Yin Y.L., Liu G., Yu X.L., Li Y.H., Yang G., Li T.J., Wu G.Y. (2012). Effect of Dietary Arginine Supplementation on Reproductive Performance of Mice with Porcine Circovirus Type 2 Infection. Amino Acids.

[7] Elango R., Ball R.O., Pencharz P.B. (2009). Amino Acid Requirements in Humans: With a Special Emphasis on the Metabolic Availability of Amino Acids. Amino Acids.

[8] Bodis J., Farkas B., Nagy B., Kovacs K., Sulyok E. (2022). The Role of L-Arginine-NO System in Female Reproduction: A Narrative Review. Int. J. Mol. Sci..

[9] Wu G., Bazer F.W., Burghardt R.C., Johnson G.A., Kim S.W., Li X.L., Satterfield M.C., Spencer T.E. (2010). Impacts of Amino Acid Nutrition on Pregnancy Outcome in Pigs: Mechanisms and Implications for Swine Production. J. Anim. Sci..

[10] Li P., Knabe D.A., Kim S.W., Lynch C.J., Hutson S.M., Wu G. (2009). Lactating Porcine Mammary Tissue Catabolizes Branched-Chain Amino Acids for Glutamine and Aspartate Synthesis. J. Nutr..

[11] Steeves T.E., Gardner D.K. (1999). Temporal and Differential Effects of Amino Acids on Bovine Embryo Development in Culture. Biol. Reprod..

[12] Mantovani A., Locati M. (2013). Tumor-Associated Macrophages as a Paradigm of Macrophage Plasticity, Diversity, and Polarization: Lessons and Open Questions. Arterioscler. Thromb. Vasc. Biol..

[13] Greene J.M., Dunaway C.W., Bowers S.D., Rude B.J., Feugang J.M., Ryan P.L. (2012). Dietary L-Arginine Supplementation during Gestation in Mice Enhances Reproductive Performance and Vegfr2 Transcription Activity in the Fetoplacental Unit. J. Nutr..

[14] Halloran K.M., Stenhouse C., Wu G.Y., Bazer F.W. (2021). Arginine, Agmatine, and Polyamines: Key Regulators of Conceptus Development in Mammals. Adv. Exp. Med. Biol..

[15] Cai X., Shang L., Li Y.S., Cao Y., Shi F. (2026). Arginine Transporters in Human Cancers: Emerging Mechanisms and Clinical Implications. Biomolecules.

[16] Wang X.Q., Frank J.W., Little D.R., Dunlap K.A., Satterfield M.C., Burghardt R.C., Hansen T.R., Wu G., Bazer F.W. (2014). Functional Role of Arginine during the Peri-Implantation Period of Pregnancy. I. Consequences of Loss of Function of Arginine Transporter SLC7A1 mRNA in Ovine Conceptus Trophectoderm. FASEB J..

[17] Liu B.M., Duan L.K., Liu X.J., Bazer F.W., Wang X.Q. (2025). Uterine Histotroph and Conceptus Development. IV. Metabolomic Analyses of Uterine Luminal Fluid Reveals Regulatory Landscapes during the Peri-Implantation Period of Pregnancy in Pigs†. Biol. Reprod..

[18] Zeng X.F., Mao X.B., Huang Z.M., Wang F.L., Wu G.Y., Qiao S.Y. (2013). Arginine Enhances Embryo Implantation in Rats through PI3K/PKB/mTOR/NO Signaling Pathway during Early Pregnancy. Reproduction.

[19] Caldwell R.W., Rodriguez P.C., Toque H.A., Narayanan S.P., Caldwell R.B. (2018). Arginase: A Multifaceted Enzyme Important in Health and Disease. Physiol. Rev..

[20] Niu F.L., Yu Y., Li Z.Z., Ren Y.Y., Li Z., Ye Q., Liu P., Ji C.S., Qian L., Xiong Y. (2022). Arginase: An Emerging and Promising Therapeutic Target for Cancer Treatment. Biomed. Pharmacother..

[21] Jenkinson C.P., Grody W.W., Cederbaum S.D. (1996). Comparative Properties of Arginases. Comp. Biochem. Physiol. B Biochem. Mol. Biol..

[22] Caldwell R.B., Toque H.A., Narayanan S.P., Caldwell R.W. (2015). Arginase: An Old Enzyme with New Tricks. Trends Pharmacol. Sci..

[23] Li H., Meininger C.J., Hawker J.R., Haynes T.E., Kepka-Lenhart D., Mistry S.K., Morris S.M., Wu G. (2001). Regulatory Role of Arginase I and II in Nitric Oxide, Polyamine, and Proline Syntheses in Endothelial Cells. Am. J. Physiol. Endocrinol. Metab..

[24] Pandey D., Bhunia A., Oh Y.J., Chang F., Bergman Y., Kim J.H., Serbo J., Boronina T.N., Cole R.N., Van Eyk J. (2014). OxLDL Triggers Retrograde Translocation of Arginase2 in Aortic Endothelial Cells via ROCK and Mitochondrial Processing Peptidase. Circ. Res..

[25] Pudlo M., Demougeot C., Girard-Thernier C. (2017). Arginase Inhibitors: A Rational Approach Over One Century. Med. Res. Rev..

[26] Lefèvre P.L.C., Palin M.-F., Murphy B.D. (2011). Polyamines on the Reproductive Landscape. Endocr. Rev..

[27] Kang B., Wang X., An X.G., Ji C.M., Ling W.K., Qi Y.X., Li S., Jiang D.M. (2023). Polyamines in Ovarian Aging and Disease. Int. J. Mol. Sci..

[28] Zhang Y., Bai J., Cui Z.K., Li Y., Gao Q., Miao Y.L., Xiong B. (2023). Polyamine Metabolite Spermidine Rejuvenates Oocyte Quality by Enhancing Mitophagy during Female Reproductive Aging. Nat. Aging.

[29] Tajima M., Harada T., Ishikawa T., Iwahara Y., Kubota T. (2012). Augmentation of Arginase II Expression in the Human Endometrial Epithelium in the Secretory Phase. J. Med. Dent. Sci..

[30] Han I.H., Choi I., Kim S., Kwon M., Choi H., Bae H. (2025). Immunomodulatory Peptide-Drug Conjugate MEL-dKLA Suppresses Progression of Prostate Cancer by Eliminating M2-like Tumor-Associated Macrophages. Front. Immunol..

[31] Moschetti G., Oliveri D., De Matteis V., Zaccaria M., Rondelli D., Griego A., Scarpa E., Rizzello L. (2025). A Critical Guideline for Controlling Monocyte-Derived Macrophages Phenotypes. Front. Immunol..

[32] Aikawa S., Deng W., Liang X., Yuan J., Bartos A., Sun X., Dey S.K. (2020). Uterine Deficiency of High-Mobility Group Box-1 (HMGB1) Protein Causes Implantation Defects and Adverse Pregnancy Outcomes. Cell Death Differ..

[33] Ogino K.K., Kubo M., Takahashi H., Zhang R., Zou Y., Fujikura Y. (2013). Anti-Inflammatory Effect of Arginase Inhibitor and Corticosteroid on Airway Allergic Reactions in a Dermatophogoides Farinae-Induced NC/Nga Mouse Model. Inflammation.

[34] Pacheco J.H.L., Elizondo G. (2023). Interplay between Estrogen, Kynurenine, and AHR Pathways: An Immunosuppressive Axis with Therapeutic Potential for Breast Cancer Treatment. Biochem. Pharmacol..

[35] Zhou M.L., Xu H.H., Zhang D., Si C.C., Zhou X.W., Zhao H., Liu Q., Xu B.F., Zhang A.J. (2021). Decreased PIBF1/IL6/p-STAT3 during the Mid-Secretory Phase Inhibits Human Endometrial Stromal Cell Proliferation and Decidualization. J. Adv. Res..

[36] Wang P.K., Du S.L., Guo C.H., Ni Z.L., Huang Z.Y., Deng N., Bao H., Deng W., Lu J., Kong S. (2024). The Presence of Blastocyst within the Uteri Facilitates Lumenal Epithelium Transformation for Implantation via Upregulating Lysosome Proteostasis Activity. Autophagy.

[37] Thapa R., Monsivais D. (2026). Mechanisms Driving Resistance to Oxidative Stress during Endometrial Stromal Cell Decidualization†. Biol. Reprod..

[38] Chang C., Cheng Y.-Y., Kamlapurkar S., White S., Tang P.W., Elhaw A.T., Javed Z., Aird K.M., Mythreye K., Phaëton R. (2024). GPX3 Supports Ovarian Cancer Tumor Progression in Vivo and Promotes Expression of GDF15. Gynecol. Oncol..

[39] Ousman S.S., Tomooka B.H., van Noort J.M., Wawrousek E.F., O’Connor K.C., Hafler D.A., Sobel R.A., Robinson W.H., Steinman L. (2007). Protective and Therapeutic Role for alphaB-Crystallin in Autoimmune Demyelination. Nature.

[40] Qiu Y.Q., Yang X.F., Wang L., Gao K.G., Jiang Z.Y. (2019). L-Arginine Inhibited Inflammatory Response and Oxidative Stress Induced by Lipopolysaccharide via Arginase-1 Signaling in IPEC-J2 Cells. Int. J. Mol. Sci..

[41] Shi O., Morris S.M., Zoghbi H., Porter C.W., O’Brien W.E. (2001). Generation of a Mouse Model for Arginase II Deficiency by Targeted Disruption of the Arginase II Gene. Mol. Cell. Biol..

[42] Wong E.S.W., Man R.Y.K., Ng K.F.J., Leung S.W.S., Vanhoutte P.M. (2018). L-Arginine and Arginase Products Potentiate Dexmedetomidine-Induced Contractions in the Rat Aorta. Anesthesiology.

[43] Ayers-Ringler J., Kolumam Parameswaran P., Khashim Z., Dai D., Ding Y.-H., Kallmes D.F., Kadirvel R. (2022). L-Arginine Reduces Downstream Vascular Contractility after Flow-Diverting Device Deployment: A Preliminary Study in a Rabbit Model. Interv. Neuroradiol..

[44] Yu Y.Q., Liu Y.Y., Sui X.S., Sui Y.Y., Wang Z., Mendelson C.R., Gao L. (2023). Arginase 1 and L-Arginine Coordinate Fetal Lung Development and the Initiation of Labor in Mice. EMBO Rep..

[45] Sun X.F., Park C.B., Deng W.B., Potter S.S., Dey S.K. (2016). Uterine Inactivation of Muscle Segment Homeobox (Msx) Genes Alters Epithelial Cell Junction Proteins during Embryo Implantation. FASEB J..

[46] Cheng J.G., Chen J.R., Hernandez L., Alvord W.G., Stewart C.L. (2001). Dual Control of LIF Expression and LIF Receptor Function Regulate Stat3 Activation at the Onset of Uterine Receptivity and Embryo Implantation. Proc. Natl. Acad. Sci. USA.

[47] Vasquez-Dunddel D., Pan F., Zeng Q., Gorbounov M., Albesiano E., Fu J., Blosser R.L., Tam A.J., Bruno T., Zhang H. (2013). STAT3 Regulates Arginase-I in Myeloid-Derived Suppressor Cells from Cancer Patients. J. Clin. Investig..

[48] Zeng X.F., Wang F.L., Fan X., Yang W.J., Zhou B., Li P.F., Yin Y.L., Wu G.Y., Wang J. (2008). Dietary Arginine Supplementation during Early Pregnancy Enhances Embryonic Survival in Rats. J. Nutr..

[49] Wu G.Y., Bazer F.W., Satterfield M.C., Li X., Wang X.Q., Johnson G.A., Burghardt R.C., Dai Z., Wang J., Wu Z. (2013). Impacts of Arginine Nutrition on Embryonic and Fetal Development in Mammals. Amino Acids.

[50] Li X.L., Bazer F.W., Johnson G.A., Burghardt R.C., Erikson D.W., Frank J.W., Spencer T.E., Shinzato I., Wu G. (2010). Dietary Supplementation with 0.8% L-Arginine between Days 0 and 25 of Gestation Reduces Litter Size in Gilts. J. Nutr..

[51] Krotova K., Patel J.M., Block E.R., Zharikov S. (2010). Hypoxic Upregulation of Arginase II in Human Lung Endothelial Cells. Am. J. Physiol. Cell Physiol..

[52] Matsumoto L., Hirota Y., Saito-Fujita T., Takeda N., Tanaka T., Hiraoka T., Akaeda S., Fujita H., Shimizu-Hirota R., Igaue S. (2018). HIF2α in the Uterine Stroma Permits Embryo Invasion and Luminal Epithelium Detachment. J. Clin. Investig..

[53] Andrianifahanana M., Moniaux N., Batra S.K. (2006). Regulation of Mucin Expression: Mechanistic Aspects and Implications for Cancer and Inflammatory Diseases. Biochim. Biophys. Acta.

[54] Paria B.C., Ma W., Tan J., Raja S., Das S.K., Dey S.K., Hogan B.L. (2001). Cellular and Molecular Responses of the Uterus to Embryo Implantation Can Be Elicited by Locally Applied Growth Factors. Proc. Natl. Acad. Sci. USA.

[55] Hsiao K.Y., Chang N., Tsai J.L., Lin S.C., Tsai S.J., Wu M.H. (2017). Hypoxia-Inhibited DUSP2 Expression Promotes IL-6/STAT3 Signaling in Endometriosis. Am. J. Reprod. Immunol..

[56] El-Sherbiny H.R., Samir H., El-Shalofy A.S., Abdelnaby E.A. (2022). Exogenous L-Arginine Administration Improves Uterine Vascular Perfusion, Uteroplacental Thickness, Steroid Concentrations and Nitric Oxide Levels in Pregnant Buffaloes under Subtropical Conditions. Reprod. Domest. Anim. Zuchthyg..

[57] Tian X.C., Wang Q.Y., Li D.D., Wang S.T., Yang Z.Q., Guo B., Yue Z.P. (2013). Differential Expression and Regulation of Cryab in Mouse Uterus during Preimplantation Period. Reproduction.

[58] Xu X., Leng J.Y., Gao F., Zhao Z.A., Deng W.B., Liang X.H., Zhang Y.J., Zhang Z.R., Li M., Sha A.G. (2014). Differential Expression and Anti-Oxidant Function of Glutathione Peroxidase 3 in Mouse Uterus during Decidualization. FEBS Lett..

[59] Zuo R.J., Zhao Y.C., Lei W., Wang T.S., Wang B.C., Yang Z.M. (2014). Crystallin αB Acts as a Molecular Guard in Mouse Decidualization: Regulation and Function during Early Pregnancy. FEBS Lett..

[60] Ma W., Song H., Das S.K., Paria B.C., Dey S.K. (2003). Estrogen Is a Critical Determinant That Specifies the Duration of the Window of Uterine Receptivity for Implantation. Proc. Natl. Acad. Sci. USA.

[61] Li Y., Chen S.T., He Y.Y., Li B., Yang C., Yang Z.S., Yang Z.M. (2023). The Regulation and Function of Acetylated High-Mobility Group Box 1 during Implantation and Decidualization. Front. Immunol..

[62] Chen S.T., Shi W.W., Ran F., Liu C.K., Luo H.N., Wu L.J., Wu Y., Zhang T.T., Yang Z.M. (2024). The Activation of cGAS-STING Pathway Causes Abnormal Uterine Receptivity in Aged Mice. Aging Cell.

[63] Liang Y.X., Liu L., Jin Z.Y., Liang X.H., Fu Y.S., Gu X.W., Yang Z.M. (2018). The High Concentration of Progesterone Is Harmful for Endometrial Receptivity and Decidualization. Sci. Rep..

[64] Luo H.N., Yang H.Y., Wang Z.M., Luo J.M., Zhang T.T., Yang Z.M. (2025). Excessive Progesterone Impairs Mouse Decidualization via the Kyn-AhR Pathway. Front. Cell Dev. Biol..

[65] Kimura F., Takakura K., Takebayashi K., Ishikawa H., Kasahara K., Goto S., Noda Y. (2001). Messenger Ribonucleic Acid for the Mouse Decidual Prolactin Is Present and Induced during in Vitro Decidualization of Endometrial Stromal Cells. Gynecol. Endocrinol..

[66] Yang H.Y., Luo H.N., Wang Z.M., Jin D.D., Yang Z.M. (2025). Effects of Acrylamide on Mouse Implantation and Decidualization. Int. J. Mol. Sci..

[67] Rozman I., Gallo-Cordova A., Del Puerto Morales M., A Morales Ovalle M., Goya G.F., Kološa K., Hočevar D., Žegura B., Štern A. (2026). Safety of Ferrite Nanoparticles for Biomedical Applications: Cyto- and Genotoxic Effects of MxFe3-xO4 (M = Fe, Zn, Mn) in an Advanced 3D Human Hepatic in Vitro Model. Biomed. Pharmacother..

[68] Qi W.M., Zhao T., Liu M., Shi X.J., Yang Y.Q., Huang Y.Y., Li N.S., Ai K.L., Huang Q. (2025). Engineered Tantalum Sulfide Nanosheets for Effective Acute Liver Injury Treatment by Regulating Oxidative Stress and Inflammation. J. Colloid Interface Sci..

[69] Li M.Y., Wu Y., Tang H.L., Wang Y., Li B., He Y.-Y., Yan G.J., Yang Z.M. (2024). Embryo-Derived Cathepsin B Promotes Implantation and Decidualization by Activating Pyroptosis. Adv. Sci..

